# Through Reducing ROS Production, IL-10 Suppresses Caspase-1-Dependent IL-1β Maturation, thereby Preventing Chronic Neuroinflammation and Neurodegeneration

**DOI:** 10.3390/ijms21020465

**Published:** 2020-01-11

**Authors:** Yun Gao, Dezhen Tu, Ru Yang, Chun-Hsien Chu, Jau-Shyong Hong, Hui-Ming Gao

**Affiliations:** 1 MOE Key Laboratory of Model Animal for Disease Study, Model Animal Research Center, Institute for Brain Sciences, Nanjing University, Nanjing 210061, China; yun.gao@nih.gov (Y.G.); dezhen.tu@nih.gov (D.T.); yangru@nicemice.cn (R.Y.); 2 Laboratory of Neurobiology, Division of Intramural Research, National Institute of Environmental Health Sciences/National Institutes of Health, Research Triangle Park, NC 27709, USA; chunhsienchu@gmail.com (C.-H.C.); hong3@niehs.nih.gov (J.-S.H.)

**Keywords:** IL-10, NLRP3 inflammasome, IL-1β, neuroinflammation, ROS, Parkinson’s disease

## Abstract

Chronic neuroinflammation contributes to the pathogenesis of Parkinson’s disease (PD). However, cellular and molecular mechanisms by which chronic neuroinflammation is formed and maintained remain elusive. This study aimed to explore detailed mechanisms by which anti-inflammatory cytokine interleukin-10 (IL-10) prevented chronic neuroinflammation and neurodegeneration. At 24 h after an intranigral injection of lipopolysaccharide (LPS), levels of NLRP3, pro-caspase-1, pro-IL-1β, active caspase-1, and mature IL-1β in the midbrain were much higher in IL-10^−/−^ mice than wildtype mice. Mechanistically, IL-10^−/−^ microglia produced more intracellular reactive oxygen species (iROS) and showed more profound activation of NADPH oxidase (NOX2) than wildtype microglia. Meanwhile, suppression of NOX2-derived iROS production blocked LPS-elicited caspase-1 activation and IL-1β maturation in IL-10^−/−^ microglia in vitro and in vivo. One month after intranigral LPS injection, IL-10^−/−^ mice revealed more profound microglial activation and dopaminergic neurodegeneration in the substantia nigra than wildtype mice. Importantly, such PD-like pathological changes were prevented by IL-1β neutralization. Collectively, IL-10 inhibited LPS-elicited production of NOX2-derived iROS thereby suppressing synthesis of NLRP3, pro-caspase-1 and pro-IL-1β and their activation and cleavage. By this mechanism, IL-10 prevented chronic neuroinflammation and neurodegeneration. This study suggested boosting anti-inflammatory effects of IL-10 and suppressing NLRP3 inflammasome activation could be beneficial for PD treatment.

## 1. Introduction

Parkinson’s disease (PD), the second most common neurodegenerative disease, is characterized by progressive loss of dopamine (DA) neurons in the substantia nigra (SN) and motor dysfunction. Emerging evidence has indicated important contributions of chronic neuroinflammation to PD pathogenesis [1,2,3,4,5,6]. However, how chronic neuroinflammation is formed and maintained remains elusive and warrants further investigation. Cytokine interleukin-10 (IL-10) is best known by its counter-regulatory role in an attempt to resolve inflammation [7]. Actually, IL-10 is a pleiotropic regulatory cytokine with both immune-suppressive and immune-stimulatory properties. Beneficial anti-inflammatory effects of IL-10 have been shown in animal models of neurodegenerative diseases, such as Alzheimer’s disease, PD, and multiple sclerosis [7,8,9,10,11,12]. Surprisingly, a stimulatory role of IL-10 in antibody production has been found to be associated with the pathogenesis of multiple sclerosis [13]. Therefore, the precise role and its underlying mechanism of IL-10 in neurodegenerative diseases, including PD, needs further investigation. 

Recent studies have indicated that IL-10 suppresses activation of NOD-like receptor pyrin domain containing 3 (NLRP3) inflammasome triggered by ATP, Alum, or monosodium urate crystal in lipopolysaccharide (LPS) primed peripheral macrophages [14]. Multiple studies have implicated important roles of activation of NLRP3 inflammasome in PD [15,16]. However, the molecular mechanism of IL-10-mediated inhibition of NLRP3 inflammasome activation in brain microglia remains unclear. This study elucidated that IL-10 inhibited LPS-elicited production of NADPH oxidase (NOX2)-derived intracellular reactive oxygen species (iROS) thereby suppressing synthesis of NLRP3, pro-caspase-1, and pro-IL-1β, as well as their activation and cleavage. This study demonstrated an important mechanism for IL-10 to downregulate NLRP3 inflammasome activation and caspase-1-dependent IL-1β maturation, thereby, preventing chronic neuroinflammation and dopaminergic neurodegeneration. 

## 2. Results

### 2.1. IL-10 Suppressed LPS-Elicited Synthesis of NLRP3, Pro-Caspase-1, and Pro-IL-1β, as Well as Their Activation and Cleavage in Brain Microglia

**Figure 1:**
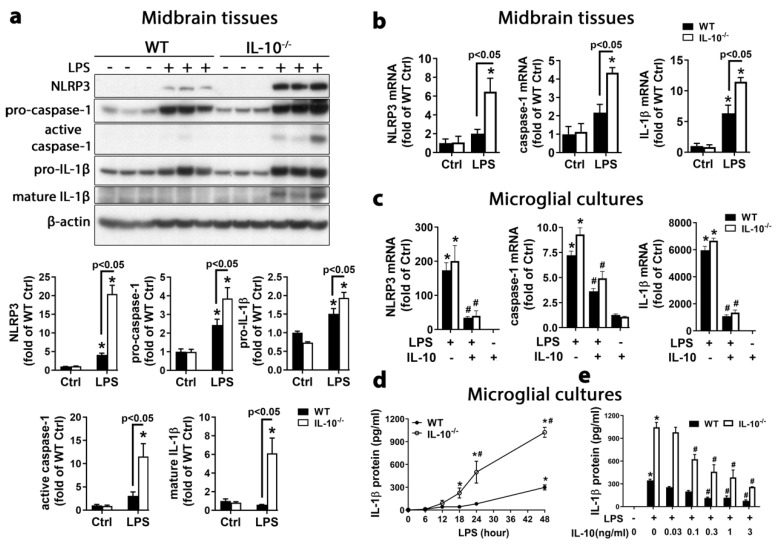
IL-10 suppressed expression, as well as activation and cleavage of NLRP3, pro-caspase-1, and pro-IL-1β. (**a**) Immunoblot analysis showed greater upregulation of NLRP3, pro-caspase-1 p45, pro-IL-1β p31 and much higher levels of active caspase-1 p20 and mature IL-1β p17 in midbrains of IL-10^−/−^ mice than wildtype (WT) mice at 24 h after an intranigral injection of lipopolysaccharide (LPS) (3 μg). (**b**) The qPCR analysis showed much higher levels of mRNA of NLRP3, caspase-1, and IL-1β in the midbrain of IL-10^−/−^ mice than WT mice at 6 h after intranigral LPS injection. (**c**) The qPCR analysis revealed similar upregulation of mRNA expression of NLRP3, caspase-1, and IL-1β in WT and IL-10^−/−^ microglia-enriched cultures at 6 h after LPS treatment; such mRNA upregulation was dramatically diminished by pretreatment for 30 min with recombinant IL-10. (**d**) ELISA showed much more IL-1β release in LPS-treated IL-10^−/−^ mixed-glia cultures than WT cultures. (**e**) ELISA detected attenuation of LPS-elicited IL-1β release by 9 h post treatment with recombinant IL-10 protein in IL-10^−/−^ microglia-enriched cultures at 48 h after LPS treatment. Results represent 3 to 4 independent experiments. Significance was determined by two-way ANOVA with Sidak’s multiple comparisons for a, b and d and with Tukey’s multiple comparisons for c and e. * *p* < 0.05 as compared with the corresponding saline-treated control, # *p* < 0.05 as compared with the corresponding LPS-treated cultures (**c**,**e**) and # *p* < 0.05 as compared with corresponding WT groups (**d**). Ctrl, control.

It is well established that activation of NLRP3 inflammasome and caspase-1 triggers cleavage of proinflammatory cytokines interleukin-1β (IL-1β) and IL-18 leading to their secretion into extracellular space. To examine how IL-10 regulates NLRP3 inflammasome and, consequently, affects PD pathogenesis, we stereotaxically injected LPS into the SN of wildtype (WT) and IL-10^−/−^ mice to generate an in vivo PD model. At 24 h after LPS injection, we examined activation of NLRP3 inflammasome and cleavage of pro-caspase-1 and pro-IL-1β in both mice. As shown in Figure 1a, levels of NLRP3, pro-caspase-1, pro-IL-1β, active caspase-1 p20, and mature IL-1β p17 in the midbrain were much higher in IL-10^−/−^ mice than WT mice. Compared with the WT mice, IL-10^−/−^ mice showed much higher mRNA levels of NLRP3, caspase-1, and IL-1β in midbrains at 6 h after intranigral LPS injection (Figure 1b). Pretreatment of WT and IL-10^−/−^ microglia-enriched cultures with recombinant IL-10 protein 30 min before LPS treatment dramatically reduced mRNA transcripts of NLRP3, caspase-1, and IL-1β, which was measured at 6 h after LPS treatment (Figure 1c). Thus, both in vivo and in vitro findings indicated that IL-10 downregulated NLRP3, caspase-1, and IL-1β (Figure 1a–c). No significant difference in mRNA levels of NLRP3, caspase-1, and IL-1β between WT and IL-10^−/−^ microglia-enriched cultures after LPS treatment (Figure 1c) was due to delayed IL-10 production in WT microglial culture system after LPS treatment [17,18,19,20]. In other words, delayed production of IL-10 in WT microglial cultures made IL-10 miss the window to affect gene transcription and mRNA degradation of these genes, which happened earlier. 

IL-10^−/−^ mixed-glia cultures secreted much more IL-1β than that of WT cultures after LPS stimulation, and IL-1β secretion occurred much earlier in IL-10^−/−^ cultures than WT cultures. At 18 and 48 h after LPS treatment, supernatant IL-1β p17 in IL-10^−/−^ and WT cultures was significantly increased, respectively (Figure 1d). Post treatment with recombinant IL-10 protein at 9 h after LPS treatment significantly attenuated IL-1β secretion 48 h after LPS treatment in microglia-enriched cultures in a dose-dependent manner (Figure 1e). This post-treatment paradigm allowed us to specifically investigate how IL-10 regulated NLRP3 inflammasome activation and IL-1β maturation with minified influence on gene transcription and mRNA degradation, which typically occurred and peaked within hours after LPS treatment. The in vivo and in vitro results together indicated that IL-10 was able to mitigate NLRP3 inflammasome activation and IL-1β maturation and secretion in brain microglia through downregulating synthesis of NLRP3, pro-caspase-1, and pro-IL-1β, and inhibiting their activation and cleavage. 

### 2.2. Inhibition of Activation of NLRP3 Inflammasome and Caspase-1 Blocked IL-1β Maturation in Microglial Cultures and Mouse Midbrains after LPS Administration

**Figure 2:**
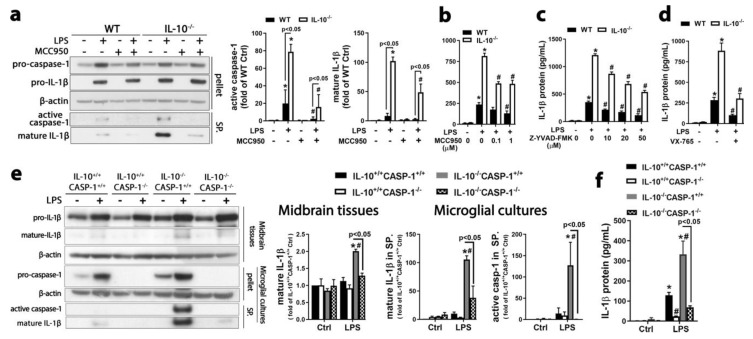
IL-10 suppressed NLRP3 inflammasome-dependent caspase-1 activation and IL-1β secretion. (**a**–**d**) At 48 h after LPS treatment of microglia-enriched cultures, immunoblot analysis (**a**) and ELISA (**b**–**d**) revealed attenuation of LPS-elicited caspase-1 activation and IL-1β release in IL-10^−/−^ microglia by post treatment with MCC950, Z-YVAD-FMK, or VX-765 (10 μM). (**e**) Immunoblot analysis detected more mature IL-1β in the midbrain and microglia-enriched cultures of IL-10^−/−^/caspase-1^+/+^ than IL-10^+/+^/caspase-1^+/+^ at 24 h after intranigral LPS injection and 48 h after LPS treatment, respectively. Genetic deletion of caspase-1 prevented LPS-elicited cleavage of pro-IL-1β in IL-10^−/−^/caspase-1^−/−^ mice (*n* = 3 mice per group) and microglia-enriched cultures (3 independent experiments). (**f**) ELISA indicated that genetic ablation of caspase-1 blunted LPS-elicited IL-1β release in IL-10^+/+^/caspase-1^−/−^ and IL-10^−/−^/caspase-1^−/−^ mixed-glia cultures 48 h after LPS treatment. Results are the mean ± SEM of 3 independent experiments, and significance was determined by two-way ANOVA with Tukey’s multiple comparisons. * *p* < 0.05 as compared with the corresponding saline-treated control; # *p* < 0.05 as compared with the corresponding LPS-treated cultures in a–d and # *p* < 0.05 as compared with the corresponding LPS-injected IL-10^+/+^caspase-1^+/+^ mice or LPS-treated IL-10^+/+^caspase-1^+/+^ cultures in e and f. CASP-1, caspase-1.

As described above, a significant increase in supernatant IL-1β p17 occurred at 18 h after LPS treatment in IL-10^−/−^ mixed-glia cultures but occurred much later in WT mixed-glia cultures (Figure 1d). Unless otherwise indicated, the post-treatment paradigm with a 9 h delay after LPS treatment was used in the following in vitro experiments studying mechanisms of IL-10’s regulation on NLRP3 inflammasome activation and IL-1β maturation. This post-treatment paradigm better balanced decay of various reagents used in these experiments and their effective duration. Post treatment with selective NLRP3 inhibitor MCC950 (a small molecule with a half-life of 3.27 h after a single dosing in mice) significantly attenuated LPS-elicited cleavage of pro-caspase-1 and pro-IL-1β, as well as IL-1β release in microglia-enriched cultures (Figure 2a,b). Z-YVAD-FMK (a cell-permeable, irreversible inhibitor of caspase-1) and VX-765 (a selective inhibitor of caspase-1/11) suppressed LPS-elicited IL-1β release (Figure 2c,d). We next cross-bred IL-10^−/−^ mice with caspase-1^−/−^ mice and generated IL-10^+/+^/caspase-1^+/+^, IL-10^−/−^/caspase-1^+/+^, IL-10^+/+^/caspase-1^−/−^, and IL-10^−/−^/caspase-1^−/−^ mice. At 24 h after intranigral LPS injection, IL-10^−/−^/caspase-1^+/+^ mice revealed more mature IL-1β in the midbrain than IL-10^+/+^/caspase-1^+/+^ mice (Figure 2e). Similarly, at 48 h after LPS treatment, microglia-enriched (Figure 2e) and mixed-glia cultures (Figure 2f) prepared from IL-10^−/−^/caspase-1^+/+^ mice released more mature IL-1β into the culture medium than the cultures prepared from IL-10^+/+^/caspase-1^+/+^ mice. More importantly, genetic deletion of caspase-1 prevented LPS-elicited cleavage of pro-IL-1β in midbrains and extracellular release of mature IL-1β (Figure 2e,f). Collectively, IL-10 regulated cleavage of pro-IL-1β through modulating NLRP3 inflammasome-dependent caspase-1 activation.

### 2.3. IL-10 Inhibited iROS Production to Suppress NLRP3 Inflammasome Activation and IL-1β Maturation

**Figure 3:**
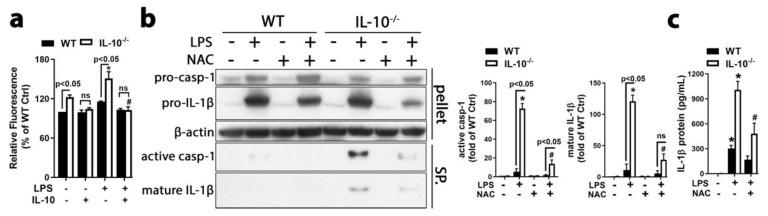
More accumulated ROS in IL-10^−/−^ microglia mediated NLRP3 inflammasome-dependent caspase-1 activation and IL-1β secretion elicited by LPS. (**a**) Quantification of fluorescent intensity of fluorescent probe CM-H_2_-DCFDA showing more production of iROS in IL-10^−/−^ microglia-enriched cultures than WT microglia treated with LPS for 18 h. Pretreatment for 30 min with recombinant IL-10 blocked LPS-induced iROS production in IL-10^−/−^ microglia. (**b**,**c**) Pretreatment of microglia-enriched (**b**) and mix-glia cultures (**c**) with ROS scavenger NAC for 30 min blocked cleavage of pro-caspase-1 and pro-IL-1β (**b**) and IL-1β release (**b**,**c**) detected at 48 h after LPS treatment. Results are the mean ± SEM, and significance was determined by two-way ANOVA with Tukey’s multiple comparisons for a (*n* = 2), b and c (*n* = 3). * *p* < 0.05 as compared with the corresponding vehicle-treated control and # *p* < 0.05 as compared with the corresponding LPS-treated groups. CASP-1, caspase-1.

In many in vitro studies, priming by LPS or Pam3CSK4 (a ligand for toll-like receptor 1/2 and a potent activator of the proinflammatory transcription factor NF-κB) has been used to upregulate NLRP3, pro-caspases, and pro-IL-1β [15,21,22]. However, without priming, the NLRP3 inflammasome is activated by ATP or nigericin in human microglia [23], by fibrillar α-synuclein in mouse microglia and human monocytes [24,25,26], or by 24 h incubation with LPS alone in human peripheral blood mononuclear cells (PBMCs) seeded at high density [27]. In our study, prolonged stimulation with LPS alone was sufficient to induce activation of NLRP3 inflammasome and caspase-1, as well as extracellular release of mature IL-1β at low levels in WT microglia and much higher levels in IL-10^−/−^ microglia in vitro and in vivo (Figure 1a,d,e and Figure 2). It is plausible that inflammatory mediator(s) or damage-associated molecular patterns (DAMPs) produced by microglia after LPS challenge could serve as direct stimulator(s) to activate intracellular NLRP3 inflammasome. Recently, ROS have been reported to promote NLRP3 inflammasome assembly [28]. To determine whether ROS participate in differential inflammasome activation and IL-1β maturation in the presence and absence of IL-10, we first compared the iROS level in WT and IL-10^−/−^ microglia-enriched cultures after LPS stimulation. The result showed that IL-10^−/−^ microglia produced much more iROS than WT microglia detected by fluorescent probe 5-(and-6)-chloromethyl-2’,7’-dichlorodihydrofluorescein diacetate (CM-H_2_-DCFDA). Pretreatment of IL-10^−/−^ microglia for 30 min with recombinant IL-10 blocked LPS-induced iROS production (Figure 3a); moreover, pretreatment for 30 min with a commonly used ROS scavenger, N-acetyl-cysteine (NAC), significantly attenuated cleavage of pro-caspase-1 and pro-IL-1β, as well as release of mature IL-1β in LPS-treated IL-10^−/−^ microglia (Figure 3b,c). Thus, suppression of ROS production was important for IL-10-mediated blockage of LPS-elicited caspase-1 activation and IL-1β maturation. These findings not only reinforced the important role of iROS in activating NLRP3 inflammasome [21,28,29], but also identified a novel mechanisms by which IL-10 suppressed NLRP3 inflammasome activation and mature IL-1β secretion. 

**Figure 4:**
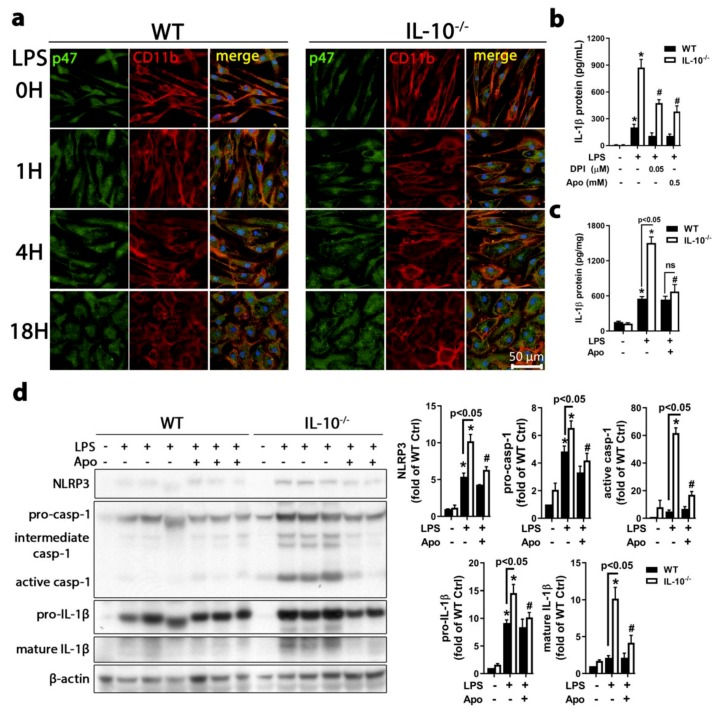
Blockage of NOX2-derived ROS inhibited synthesis and activation of NLRP3 inflammasome and consequent IL-1β maturation. (**a**) The membrane translocation of cytosolic p47 in IL-10^−/−^ microglia was detected at 1 h after LPS treatment and became more profound after prolonged treatment. LPS-treated WT microglia displayed less membrane staining of p47 as compared with time-matched IL-10^−/−^ microglia. (**b**) Pretreatment of microglia-enriched cultures with NOX2 inhibitors DPI and apocynin for 30 min significantly attenuated IL-1β release detected by ELISA at 48 h after LPS treatment. (**c**,**d**) WT and IL-10^−/−^ mice received an intranigral injection of LPS and an i.p. injection of apocynin (50 mg/kg). At 24 h after LPS injection, ELISA (**c**) indicated that apocynin abolished IL-1β elevation in midbrains of IL-10^−/−^ mice. Immunoblot analysis (**d**) revealed that upregulation of NLRP3, pro-casp-1, and pro-IL-1β, as well as cleavage of pro-caspase-1 and pro-IL-1β in the midbrain of IL-10^−/−^ mice were blocked by apocynin. Results are the mean ± SEM, and significance was determined by two-way ANOVA with Tukey’s multiple comparisons for b (*n* = 4), c (*n* = 4–5), and d (*n* = 3–4). * *p* < 0.05 as compared with the corresponding vehicle-treated control and # *p* < 0.05 as compared with the corresponding LPS-treated groups. Fluorescent images are representative of three independent experiments. Apo, apocynin and CASP-1, caspase-1.

We next examined the source of increased iROS in IL-10^−/−^ microglia after LPS stimulation. NOX2 is a major superoxide-producing enzyme complex in activated phagocytes (e.g., peripheral macrophages and brain microglia). NOX2-derived superoxide can be released into the extracellular space or phagosomes, where it forms hydrogen peroxide, peroxynitrite, or other ROSs. Hydrogen peroxide and peroxynitrite can cross the cell membrane. NOX2-derived ROS are essential for host defense and participate in cellular signaling, cell differentiation, gene expression regulation, post-translational processing of proteins, stress response, and tissue homeostasis. Its overactivation is an important pathogenic factor in chronic neuroinflammation and progressive neurodegeneration [30,31]. IL-10^−/−^ microglia showed more profound membrane translocation of cytosolic p47 (a key step in NOX2 activation) than WT microglia after LPS treatment (Figure 4a). NOX2 inhibitors, apocynin and diphenyleneiodonium (DPI), significantly attenuated LPS-elicited IL-1β release in cultured microglia (Figure 4b). More importantly, an intraperitoneal injection of apocynin blunted greater upregulation of NLRP3, pro-caspase-1, and pro-IL-1β, as well as increased cleavage of pro-caspase-1 and pro-IL-1β in midbrains of IL-10^−/−^ mice at 24 h after intranigral LPS injection (Figure 4c,d). Altogether, suppression of NOX2-derived ROS was responsible for IL-10-mediated inhibition of synthesis and activation of NLRP3 inflammasome and caspase-1 and consequent IL-1β maturation. NOX2-derived ROS are an important mediator bridging extracellular stimulation by LPS and activation of intracellular sensor NLRP3 inflammasome, which supports an “outside in” mechanism of NLRP3 inflammasome activation by extracellular stimulators beyond the well-known intracellular function and activation mechanisms of NLRP3 inflammasome. 

### 2.4. Neutralization of IL-1β Attenuated Microglial Activation and Dopaminergic Neurodegeneration

**Figure 5:**
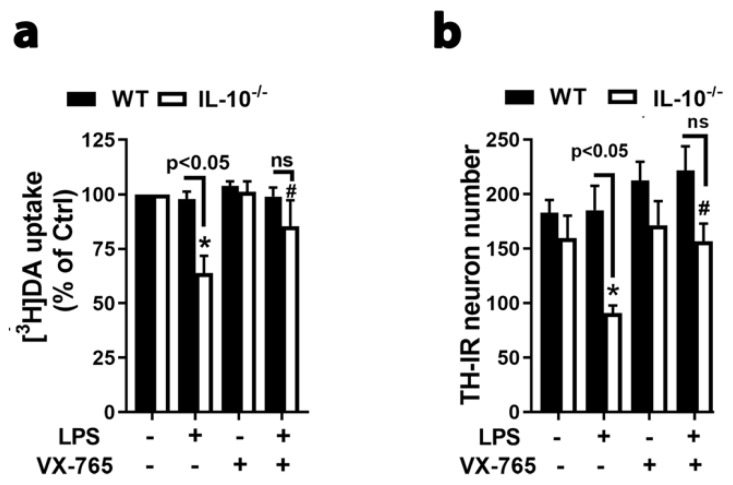
Inhibition of caspase-1 attenuated LPS-induced damages of dopamine (DA) neurons. (**a**,**b**) [^3^H]DA uptake assay (**a**) and counting of TH-IR DA neurons (**b**) conducted 7 d after LPS treatment showed dopaminergic neuroprotection by post treatment with 10 μM VX-765 with a 9 h delay in midbrain neuron-glia cultures generated from IL-10^−/−^ mice. Results are the mean ± SEM of 4 independent experiments. Significance was determined by two-way ANOVA with Tukey’s multiple comparisons (**a**) or student *t*-test (**b**). * *p* < 0.05 as compared with the corresponding saline-treated control cultures and # *p* < 0.05 as compared with the corresponding LPS-treated cultures. ns, not significant.

We, next, examined whether IL-10’s regulation on NLRP3 activation and IL-1β release affected brain inflammation and neuronal survival. The [^3^H]DA uptake assay and visual count of tyrosine hydroxylase (TH) immunoreactive (IR) neurons indicated that LPS-induced loss of DA neurons only occurred in midbrain neuron-glia cultures prepared from IL-10^−/−^ mice and was blocked by caspase-1 inhibitor VX-765 (Figure 5a,b). These findings supported important roles of caspase-1 activation in inflammation-mediated neurodegeneration and in IL-10’s regulation on NLRP3 inflammasome activation.

**Figure 6:**
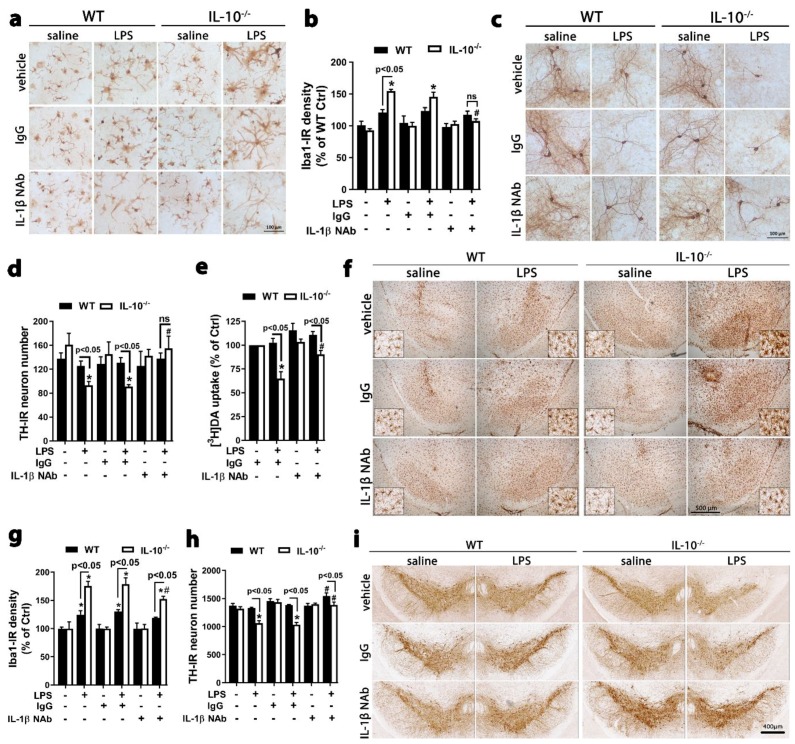
Neutralization of IL-1β attenuated microglial activation and dopaminergic neurodegeneration in IL-10^−/−^ mice and midbrain neuron-glia cultures. (**a**–**e**) Immunohistochemistry images, densitometry analysis, cell count, and [^3^H]DA uptake assay showing microglial activation (**a**,**b**) and loss of TH-IR DA neurons (**c**–**e**) 7 d after LPS treatment in midbrain neuron-glia cultures generated from IL-10^−/−^ mice. Post treatment with an 18 h delay with 1 μg/mL IL-1β Nab, but not control IgG, blocked microglial activation (**a**,**b**) and dopaminergic neurodegeneration (**c**–**e**). (**f**,**g**) Immunohistochemistry images and densitometry analysis showing more profound microglial activation in the SN, as indicated by enlarged cell size and irregular cell shape (**f**) and Iba-1 upregulation (**f**,**g**) in IL-10^−/−^ mice than WT mice one month after an intranigral injection of 3 μg LPS. Co-injection of 1μg IL-1β NAb, but not control IgG, dampened LPS-elicited microglial activation. In the SN injected with saline and IgG or saline and NAb, microglia exhibited the ramified resting morphology and only displayed activated morphology in the very limited area along the needle tracks. (**h**,**i**) Cell count and immunohistochemistry images showing significant reduction in the number of nigral TH-IR DA neurons in IL-10^−/−^ mice, but not in WT mice, one month after intranigral LPS injection. IL-1β NAb, but not control IgG, reversed such neuronal loss in IL-10^−/−^ mice. Results are the mean ± SEM. Significance was determined by two-way ANOVA with Tukey’s multiple comparisons for b (*n* = 3), e (*n* = 5), g (*n* = 4), and h (*n* = 3 to 6) and student t-test for d (*n* = 4). * *p* < 0.05 as compared with the corresponding saline-treated control cultures or saline-injected control mice and # *p* < 0.05 as compared with the corresponding LPS-treated cultures or LPS-injected mice. Immunostaining images were representative of three (**a**), four (**c,f**), and three to six (**i**) independent experiments. ns, not significant.

Moreover, LPS induced more dramatic morphological changes (e.g., enlarged cell bodies and irregular shape) and greater upregulation of ionized calcium binding adaptor 1 (Iba1) in IL-10^−/−^ midbrain neuron-glia cultures than WT cultures (Figure 6a,b). Post treatment with an 18 h delay with IL-1β neutralization antibody (NAb), but not control IgG, diminished LPS-elicited microglial activation and dopaminergic neurodegeneration in midbrain neuron-glia cultures generated from IL-10^−/−^ mice (Figure 6a–e). Notably, the post-treatment time point (i.e., 18 h after LPS treatment) was the time point when LPS-treated IL-10^−/−^ mixed-glia cultures started to release IL-1β. In other words, the level of extracellular IL-1β in LPS-treated IL-10^−/−^ mixed-glia cultures became statistically significant difference from that of saline-treated cultures, as seen in Figure 1d. Furthermore, one month after intranigral LPS injection, nigral microglia of IL-10^−/−^ mice exhibited greater Iba1 upregulation and more profound activation than microglia of WT mice. IL-1β NAb, but not control IgG, dampened microglial activation (Figure 6f,g). More importantly, IL-10^−/−^ mice, but not WT mice, showed a significant decrease in the number of nigral TH-IR DA neurons and destruction of the intricate network of TH-IR fibers one month after intranigral LPS injection (Figure 6h,i). Thus, IL-10 protected DA neurons from LPS-induced inflammatory degeneration, which is consistent with previous findings using exogenous IL-10 in midbrain neuron-glia cultures and in LPS-injected SN [9,10]. Importantly, loss of nigral DA neurons in IL-10^−/−^ mice was blocked by IL-1β NAb but not control IgG (Figure 6h,i). These findings indicated that IL-1β is a critical mediator for chronic microglial activation and dopaminergic neurodegeneration. Suppression of IL-1β cleavage by caspase-1 and its secretion is an important mechanism for anti-inflammatory and neuroprotective effects of IL-10.

## 3. Discussion

The present study demonstrated that IL-10 prevented chronic neuroinflammation through suppression of NOX2-derived iROS production thereby downregulating synthesis of NLRP3, pro-caspase-1, and pro-IL-1β, and inhibiting their activation and cleavage. IL-1β neutralization mitigated LPS-elicited microglial activation and dopaminergic neurodegeneration in in vitro and in vivo models of PD. Caspase-1 inhibition by VX765 also attenuated dopaminergic neurodegeneration in midbrain neuron-glia cultures from IL-10^−/−^ mice. This is the first demonstration that, through inhibition of NOX2-derived iROS production, IL-10 suppressed NLRP3-caspase-1-dependent IL-1β maturation and secretion, thereby preventing chronic neuroinflammation and neurodegeneration, identifying a novel mechanism for IL-10 to prevent chronic neuroinflammation and neurodegeneration. 

IL-10, best known for its anti-inflammatory effects, is essential for immune homeostasis and inflammation resolution [7,8,9,10,11,12]. In fact, IL-10 is a cytokine with pleiotropic effects in immune regulation and inflammation, displaying both immune-suppressive and immune-stimulatory properties. For example, IL-10 suppresses functions of antigen presenting cells and T cells but promotes survival, proliferation, differentiation, and antibody production of B cells [32,33,34]. Almost all innate and adaptive immune cells produce IL-10 and also serve as its targets. The conflicting and largely inconsequential effects of systemic administration of IL-10 in systemic- and organ-specific autoimmune diseases (i.e., systemic lupus erythematosus, psoriasis, type 1 diabetes, Crohn’s disease, rheumatoid arthritis, and multiple sclerosis) in preclinical and clinical studies [34,35,36] suggest possible compartmentalization of the action of IL-10. IL-10 has been shown to upregulate expression of RNA destabilizing factor tristetraprolin to promote mRNA decay of multiple proinflammatory cytokines including IL-1β [37,38]. IL-10 also downregulates IL-1β secretion through suppression of NLRP3 inflammasome activation and subsequent IL-1β maturation in periphery immune cells [39,40]. Unlike most cytokines, IL-1β is not secreted through the conventional ER-Golgi secretion pathway [41,42,43]. The present study found that IL-10 suppressed NOX2-derived iROS to block NLRP3 inflammasome activation and cleavage of caspase-1 and pro-IL-1β, thereby preventing IL-1β secretion and chronic neuroinflammation in in vitro and in vivo models of PD. Our findings also revealed a previously unrecognized regulatory mechanism of IL-1β secretion by IL-10.

The activation of NLRP3 inflammasome and caspase-1 has been shown to participate in PD pathogenesis. NLRP3 is elevated in the SN of PD patients and animal models [15,25,44]. Deletion and inhibition of NLRP3 or caspase-1 mitigates dopaminergic neuronal loss in mouse models of PD created by injection of 1-methyl-4-phenyl-1,2,3,6-tetrahydropyridine (MPTP) or 6-hydroxydopamine or transgenic overexpression of α-synuclein [15,16,44,45,46]. Our findings that IL-1β neutralization and caspase-1 inhibition mitigated LPS-elicited dopaminergic neurodegeneration (Figure 5 and Figure 6) indicated that caspapse-1 activation and IL-1β release are critical for the formation of chronic neuroinflammation and development of inflammation-related dopaminergic neurodegeneration. Our findings not only reinforced the important contribution of activation of NLRP3 inflammasome and caspase-1 to PD pathogenesis, but also uncovered an important role of secreted IL-1β in the formation and the maintenance of chronic neuroinflammation in the absence of counter-regulation by IL-10.

It has been proposed that through triggering common cellular events (e.g., potassium efflux, lysosome damage, and mitochondrial ROS production), a broad range of stimulators including danger signals (e.g., extracellular ATP and nigericin) or pathogens (e.g., viral and bacterial pathogens) indirectly activate intracellular NLRP3 inflammasome [21]. In this study, IL-10^−/−^ microglia produced much more iROS than WT microglia (Figure 3a); ROS scavenger NAC and NOX2 inhibitors diminished NLRP3 activation in LPS-treated IL-10^−/−^ microglia (Figure 3b,c and Figure 4b–d). Therefore, iROS produced after LPS challenge served as a stimulator to activate intracellular NLRP3 inflammasome in microglia. Recent studies have also shown that iROS produced in peripheral immune cells after stimulation with asbestos and silica can activate NLRP3 [21,28,29]. The attenuation of NLRP3 activation and IL-1β secretion by NOX2 inhibitors in IL-10^−/−^ microglia-enriched cultures and mouse brains (Figure 4b–d) indicated a critical role of NOX2-derived ROS in regulating NLRP3-dependent IL-1β secretion by IL-10. Indeed, over-activation of NOX2 is an important pathogenic factor in various inflammation-related chronic diseases including PD [29,30,47]. In addition to reinforcing the important role of ROS in triggering NLRP3 inflammasome activation, our findings demonstrated, for the first time, an essential role of NOX2-derived iROS in microglial secretion of IL-1β. This study is the first demonstration that IL-10 suppresses NOX2-derived iROS to block NLRP3 inflammasome activation, caspase-1 cleavage, and IL-1β maturation and secretion in brain microglia. 

In summary, this study demonstrated an important role of IL-10 in regulating microglial secretion of IL-1β both in in vitro and in vivo. IL-10 suppressed NOX2-derived ROS production to downregulate expression of NLRP3, pro-caspase-1, and pro-IL-1β and to block NLRP3-dependent cleavage of pro-caspase-1 and pro-IL-1β, thereby preventing chronic neuroinflammation. Our results revealed novel mechanisms for IL-10 to prevent chronic neuroinflammation and neurodegeneration in models of PD. This study suggests that the boost of anti-inflammatory effects of IL-10 and prevention of IL-1β secretion are promising therapeutic strategies for PD.

## 4. Materials and Methods

### 4.1. Animals

B10.129P2(B6)-IL-10^tm1Cgn^/J (IL-10 knockout or IL-10^−/−^) mice and their WT or IL-10^+/+^ control mice (C57BL/10J), as well as B6N.129S2-Casp1^tm1Flv^/J (caspase-1 knockout or CASP-1^−/−^) mice and their WT (CASP-1^+/+^) control mice were purchased from the Jackson Laboratory (Bar Harbor, ME). We cross bred IL-10^−/−^ mice with CASP-1^−/−^ mice to generate F1 hybrids. IL-10^+/+^/CASP-1^+/+^, IL-10^−/−^/CASP-1^+/+^, IL-10^+/+^/CASP-1^−/−^, and IL-10^−/−^/CASP-1^−/−^ mice were littermates from breeding F1 males and females. All genotypes are validated by the Transnetyx company (Transnetyx, Cordova, TN, USA). We humanely treated all the animals following the National Institutes of Health Guide for Care and Use of Laboratory Animals (Institute of Laboratory Animal Resources 1996) and performed procedures that were approved by the NIEHS Animal Care and Use Committee in 16 November 2017 (AP#86-21 NL). 

### 4.2. Stereotaxic Injection into the SN

All surgical procedures were performed using aseptic techniques and ketamine (90 mg/kg)/xylazine (10 mg/kg, intraperitoneal injection) anesthesia. Following anesthesia, two-month-old mice were positioned in a small-animal stereotaxic apparatus. LPS (Escherichia coli 0111:B4; Sigma) (3 μg in 2 μL of sterile normal saline) or sterile saline (2 μL) was stereotaxically injected into the right and the left side of the SN, respectively. Similarly, LPS combined with neutralizing antibody (NAb) of IL-1β (1 μg), control IgG (1 μg), or vehicle was stereotaxically injected into the right side of the SN of WT or IL-10^−/−^ mice over a period of 5 min (0.4 μL/min) under the control of a motorized microinjection pump. Sterile saline combined with IL-1β NAb, control IgG, or vehicle was injected into the left side of SN as the control. The coordinate for the injection and the collection of mouse brains and their coronal sections were described in detailed in our published protocols [48]. 

### 4.3. Primary Cell Cultures

To prepare mixed-glia cultures, brains of 1-day old IL-10^−/−^ and WT mice were dissociated by mechanical trituration [49]. Isolated brain cells, seeded in 12-well (5 × 10^5^ cells/well) plates, were cultured in DMEM/F-12 with additional 10% heat-inactivated fetal bovine serum, 50 U/mL penicillin, 2 mM l-glutamine, 50 μg/mL streptomycin, 100 μM nonessential amino acids, and 1 mM sodium pyruvate. Once reaching confluence (11 to 12 days), mixed-glia cultures, which contained 80% glial fibrillary acidic protein (GFAP)-IR astroglia and 20% Iba1-IR microglia (~1.2 × 10^5^ microglia per well of 12-well plate), were treated with vehicle or reagents, as specified in the corresponding figure legend. 

To generate microglia-enriched cultures, we dissected and dissociated the whole brains of 1-day-old mice, as described above for mixed-glia culture preparation. At 12 to 14 days, confluent mixed-glia cultures, grown in 175 cm^2^ culture flasks, were shaken to separate microglia from astrocytes. Highly enriched microglia were plated into 12-well plates (3 × 10^5^ cells/well) or 6-well plates (6 × 10^5^ cells/well). The microglia-enriched cultures were 98% pure [49]. 

We generated midbrain neuron-glia cultures from mouse midbrains at embryonic day 14 ± 0.5 [49]. Midbrain cells, dissected and dissociated with a gentle mechanical trituration, were seeded in 24-well plates (0.75 × 10^6^ cells/well) and cultured, as described previously [49]. Seven-day old cultures, which contained about 50% astroglia, 11% Iba1-IR microglia, and 39% neurons, were treated with various reagents or vehicle. 

### 4.4. High-Affinity [^3^H]DA Uptake Assay

Midbrain neuron-glia cultures were used to perform the [^3^H]DA uptake assay as previously described [50]. Selective DA transporter inhibitor GBR-12935 (20 µM) was used to block nonspecific DA uptake. Cells were collected in 750 µL 1 N NaOH after washing with ice-cold Krebs-Ringer buffer. Radioactivity was read by liquid scintillation counting machine. Nonspecific uptake in the presence of GBR-12935 was subtracted. 

### 4.5. Quantitative Polymerase Chain Reaction (qPCR)

Total RNA of cells or midbrain tissues was extracted by using the RNeasy Mini Kit (QIAGEN, Valencia, CA, USA). Quantity and quality assessment was determined by using NanoDrop 2000UV-Vis Spectrophotometer (Thermo Fisher scientific, Waltham, MA, USA). A total of 2 μg RNA was subjected to a reverse transcription reaction, and 5 μL of cDNA was amplified by qPCR analysis using SYBR-Green Master mix (Applied Biosystems, Foster City, CA, USA) in a final volume of 20 μL. The sequences of the primers were the following: NLRP3 (F: AAA GAT GAA GGA CCC ACA GTG TAA C, R: CAT TGC TTC GTA GAT AGA GGT GTG TG); caspase-1 (F: TGA AAG ACA AGC CCA AGG TGA TC, R: ACT CCT TGT TTC TCT CCA CGG C); IL-1β (F: CTG GTG TGT GAC GTT CCC ATT A, R: CCG ACA GCA CGA GGC TTT); and GAPDH (F: TTC AAC GGC ACA GTC AAG GC, R: GAC TCC ACG ACA TAC TCA GCA CC). Reaction and data analysis were performed by using the QuantStudio 6 Flex Real-Time PCR System and QuantStudio Real-Time PCR software, respectively (Applied Biosystems). The relative difference in expression between groups was presented using cycle time values normalized to GAPDH. 

### 4.6. Cytokine Measurement by ELISA

Mouse IL-1β/IL-1F2 Quantikine ELISA Kit (R&D Systems, Minneapolis, MN, USA) was used to measure the release of mature IL-1β into cell culture medium and mouse midbrain tissue lysate.

### 4.7. Gel Electrophoresis and Immunoblot Analysis

Proteins were extracted from cultured cells using cell lysis buffer (Cell Signaling Technology, Danvers, MA, USA). Mouse midbrain tissues were homogenized in RIPA buffer and sonicated. The concentration of proteins was determined with bicinchoninic acid assay kit (Thermo Fisher Scientific, Waltham, MA, USA). Protein denaturation was performed by heated to 95 °C for 10 min. Protein samples from the culture supernatant were concentrated before electrophoresis with methanol/chloroform precipitation. Briefly, four volumes of the culture supernatant were precipitated by the addition of four volumes of methanol and one volume of chloroform. The mixture was vortexed vigorously and centrifuged for 5 min at 14,000× *g*. The upper phase (the water/methanol mix on the top of the interface) was discarded, and 1 mL methanol was added to the interphase. The mixture was vortexed and centrifuged for 5 minutes at 20,000× *g*. Then, the supernatant was removed carefully and briefly air-dried the protein pellet. The pellet was resuspended in 8 M urea. All protein samples were resolved on 4% to 12% SDS-PAGE gels, and immunoblot analyses were performed using antibodies against NLRP3 (Adipogen, AG-20B-0014, 1:1000), caspase-1 (Adipogen, AG-20B-0042, 1:1000), IL-1β (Abcam, ab9722, 1:2000), and β-actin (Cell Signaling Technology, D6A8, 1:2000). Densitometry analysis of protein bands was conducted using ImageJ software and then normalized to β-actin. 

### 4.8. Immunohistochemistry, Immunocytochemistry, and Cell Count

Paraformaldehyde-fixed cell cultures or 30 μm brain sections cut with a microtome were immunostained using antibodies against Iba1 (Wako, 019-19741, 1:5000) or TH (Millipore, AB152, 1:5000) following our published protocols [48]. Immunocytochemistry images of cell cultures were recorded with a Nikon inverted microscope connected to a charge-coupled device camera (DAGE-MTI, Michigan City, IN, USA) operated through the MetaMorph software. Immunohistochemistry images of brain sections containing the SN region were scanned using Aperio digital pathology slide scanners from Leica Biosystems. Following immunostaining, of the 60 sections covering the SN, twelve evenly spaced sections were selected for visual counting of the number of nigral TH-IR DA neurons. Sets of brain sections from 3 to 6 mice in each treatment group were used. The numbers of TH-IR neurons in cell cultures were counted under a microscope (10× power) in each well of the 24-well plate. For each experiment, 2 to 4 wells/treatment condition were used for cell count, and results from three independent experiments were obtained. Counting was performed in a blind manner. 

### 4.9. Confocal Double-Label Fluorescence

Paraformaldehyde-fixed microglia-enriched cultures grown in Lab-Tek II chamber slides (Nalge Nunc, Rochester, NY, USA) were immunostained using anti-CD11b (Bio-Rad Laboratories, MCA711G, 1:10000) and anti-p47 (EMD Millipore, 07-500, 1:500) antibodies. DAPI (4′,6-diamidino-2-phenylindole) was used to counterstain the nuclei. Fluorescent images were collected using Zeiss LSM880 Laser Scanning Confocal Microscope (Carl Zeiss Microimaging Inc., Oberkochen, Germany). 

### 4.10. Measurement of iROS

WT and IL-10^−/−^ microglia-enriched cultures grown in a black bottom 96-well plate were treated with vehicle or 20 ng/mL LPS for 18 h with or without pretreatment for 30 min with recombinant mouse IL-10. The cultures were then washed twice and incubated with 5 µM CM-H_2_-DCFDA (a cell-permeant fluorescence probe; Thermo Fisher Scientific’s Corporate) for 30 min at 37 °C to measure production of iROS. After passive diffusion into cells, CM-H_2_-DCFDA is deacetylated by esterase. Subsequent oxidation by iROS yields a fluorescent adduct that is trapped inside the cells. After washing twice with phenol red-free HBSS buffer, a SpectraMax Gemini XS fluorescence microplate reader was used to read the fluorescence density (488 nm for excitation and 525 nm for emission) (Molecular Devices).

### 4.11. Statistical Analysis

Statistical analyses were performed by using GraphPad Prism 8.0 software. All results were expressed as the mean ± SEM. Two-way ANOVA was used to analyze differences among means with genotype or treatment as independent factors. When ANOVA indicated significant differences, Tukey’s multiple comparisons, Sidak’s multiple comparisons, or *t*-test was performed to evaluate pairwise differences between means as indicated in the corresponding figure legends. In all analysis, the null hypothesis was rejected at the 0.05 level.
